# Cochlear morphology in the developing inner ear of the porcine model of spontaneous deafness

**DOI:** 10.1186/s12868-018-0426-z

**Published:** 2018-05-02

**Authors:** Wei Chen, Qing-Qing Hao, Li–Li Ren, Wei Ren, Hui-sang Lin, Wei-Wei Guo, Shi-Ming Yang

**Affiliations:** 1 Department of Otolaryngology, Head & Neck Surgery, Institute of Otolaryngology, Chinese PLA General Hospital, Beijing Key Laboratory of Hearing Impairment Prevention and Treatment, Key Laboratory of Hearing Impairment Science, Chinese PLA Medical School,Ministry of Education, Beijng, China; 2 Department of Biotechnology, Dalian Medical University, Dalian, 116044 Liaoning China

**Keywords:** Stria vascularis, MITF-M mutation, Waardenburg syndrome, Porcine model

## Abstract

**Background:**

Auditory function and cochlear morphology have previously been described in a porcine model with spontaneous WS2-like phenotype. In the present study, cochlear histopathology was further investigated in the inner ear of the developing spontaneous deafness pig.

**Results:**

We found that the stria vascularis transformed into a complex tri-laminar tissue at embryonic 85 days (E85) in normal pigs, but not in the MITF^−/−^ pigs. As the neural crest (NC) of cochlea was derived by melanocytes. MITF mutation caused failure of development of melanocytes which caused a subsequent collapse of cochlear duct and deficits of the epithelium after E100. Furthermore, the spiral ganglion neurons of cochlea in the MITF^−/−^ pigs began to degenerate at postnatal 30 days (P30). Thus, our histopathological results indicated that the malformation of the stria vascularis was a primary defect in MITF^−/−^ induced WT pigs which was resulted from the loss of NC-derived melanocytes. Subsequently, the cochleae underwent secondary degeneration of the vestibular organs. As the degeneration of spiral ganglion neurons happened after P30, it suggests that WS patients should be considered as candidates for cochlear implant.

**Conclusions:**

Our porcine model of MITF-M mutation may provide a crucial animal model for cochlear implant, cell therapy in patients with congenital hereditary hearing loss.

## Background

Waardenburg syndrome (WS) is one of the most common types of syndromic sensorineural hearing loss, characterized abnormal pigmentation in heterochromia irides, depigmented patches of skin or early greying [1]. The incidence of WS is estimated about 1/42,000 and is responsible for approximately 1–5% of congenital deafness [1–3]. WS is classified into four types (WS1, WS2, WS3 and WS4) with clinical and genetic hetero-geneses caused by mutations of six candidate genes, i.e., PAX3 (OMIM #606597), MITF (OMIM #156845), EDN3 (OMIM #131242), EDNRB (OMIM #131244), SOX10 (OMIM #602229) and SNAI2 (OMIM #602150) [1, 3]. WS2 (OMIM #193510) is similar to WS1, but is absent of distopia canthorum. About 15–21% of WS2 cases are involved with mutation in MITF gene [1, 4, 5]. MITF (microphthalmia-associated transcription factor) protein, which contains a basic helix–loop–helix leucine zipper (bHLH-LZ) domain, is crucial for the development of pigmental cells [6–8]. The MITF gene contains multiple promoters consecutively followed by a different first exon and common downstream exons. It consequently encodes distinct isoforms with different N-termini and uniform C-termini where the bHLH-LZ structures are located [9, 10]. Accordingly, these MITF isoforms exhibit tissue-specific expression and functions, of which the melanocyte-specific isoforms (MITF-M) plays a critical role in development of neural crest-derived melanocytes in inner ear and optic cup-derived retinal pigment epitheliums [11–13]. It is indicated that the correct expression level of MITF-M is required for early development and migration of melanoblasts in the neural tube, as well as their differentiation into intermediate cells in the stria vascularis (SV) of the mammalian cochlea which secrete potassium ions and produce the endolymphatic potential (both are essential for hearing) [13–16].

In our previous studies, a porcine model with spontaneous WS2-like phenotype exhibits a spontaneous sensorineural deafness and depigmentation, and a de novo short insertion in the distal regulatory region of MITF-M isoform [17]. As the effects of MITF-M isoform defection on development of cochlea is still not clear, we studied the development of the cochlea, especially on the stria vascularis (SV), of this MITF-M specific mutation porcine model in order to have a better insight of the role of MITF-M in auditory system. Our results provide the first detailed record that degradation rule and schedule of SV, hair cells and SGNs in the MITF-M mutation porcine model.

## Methods

### Animals

Pigs used in this study were provided by the Animal Breeding Facility of Chongqing Academy of Animal Science (from embryonic 65 days to postnatal 1 month). The porcine model with *MITF*-*M* specific mutation was characterized by albino and heterochromia irides [16] and the wild type were used as the control group. This study was approved by the Institutional Animal Care and Use Committee of General Hospital of People’s Liberation Army (PLA).

### Specific methods of animal care-taking

All pregnant sows were housed separately. The piglets in the first month after birth were reared in the same column of the sows, and the weaned pigs were housed in separate barns. The pregnant sows were subjected to deep anesthesia, and then the embryos were obtained utilizing the cesarean section. Before the operation, these pigs were fasted for 12 h, and water was cut-off for 24 h. The animals were given an injection into the muscles of their neck with Sumianxin II at the dose of 0.1 ml/kg. Moreover, 10 min later, isofurane was introduced with a ventilator at 0.2 ml/min. At the end, the sows were over-anesthetized with increasing the most amount of inhaled anesthesia, 0.5 ml/min isofurane until breathing and the heartbeat stopped. There was no pain in the entire process.

After birth, animals were deeply anesthetized-excessively anesthetized as described above, after the termination of breathe and heartbeat, the specimens of the iliac crest were taken without pain. Animal carcasses were handed over to the management department for unified treatment approach.

Based on the National Institutes of Health Guidelines on Use of Laboratory Animals, all experiments were conducted and approved by the General Hospital of People’s Liberation Army (PLA) Committee on Animal Care (Beijing, China).

### Tissue preparations

Inner ear of pigs were collected after deep anesthetized at different developmental stages (E65, E75, E85, E100, P1, P30 and P60). Their cochleae were dissected out immediately, then perfused with 4% paraformaldehyde fixative (through a hole made in the apical turn). All the cochlear samples were immersed in 4% formalin (for light microscopy) or 2.5% glutaraldehyde (for scanning and transmission electron microscopy) overnight at 4 °C. The cochleae from older embryos or postnatal pigs were decalcified with 10% ethylene diamine tetraacetic acid (EDTA) until the bone was soft enough for sectioning. At least three pigs were used for each age group in morphological analysis.

### Morphologic analysis

Stria vascularis semi-thin sections stained in 1% toluidine blue and cochlear celloidin embedding sections stained in hematoxylin-eosin (H&E) at different stages were observed under a light microscope (Olympus).

For the transmission electron microscopy (TEM), the cochleae were washed with 0.1 M PB, post-fixed in 1% osmium tetroxide, dehydrated in a graded series of ethanol and embedded in plastic Agar 100 resin. After polymerisation, the third cochlear turn was embedded on a blank block of Agar 100 for sectioning. Then, the sections stained with toluidine blue were examined under a transmission electron microscope (PHILIPS TECNAI10).

For the scanning electron microscopy (SEM), the cochlear samples, after being washed, post-fixed and dehydrated by the same methods as for TEM, were embedded on aluminum stubs, coated with gold particles, then examined under a scanning electron microscope (JEOL JSM35C).

### Spiral ganglion cell (SGCs) counts

The number of SGCs was determined as described previously [16]. Briefly, neurons were counted in the basal turns on the same side of the modiolus. We counted ten fields of a light microscope at 400 × magnification in each H&E taining collodion section. Six cochleae were examined at P1, P30 and P60, respectively with six normal (wild type) cochleae at the same stage used as the reference.

### Statistical analysis

All data were recorded and analyzed using the statistical software SPSS19. The mean ± standard deviation (SD) of MITF^−/−^ (MT) group was contrasted with MITF^+/+^ (WT) group using the Student’s *t* test. One-way ANOVA was used to contrast whether the number of SGCs at different stages had statistic difference. A P value of 0.05 was considered significant.

## Results

### Morphology change of stria vascularis (SV)

**Fig. 1:**
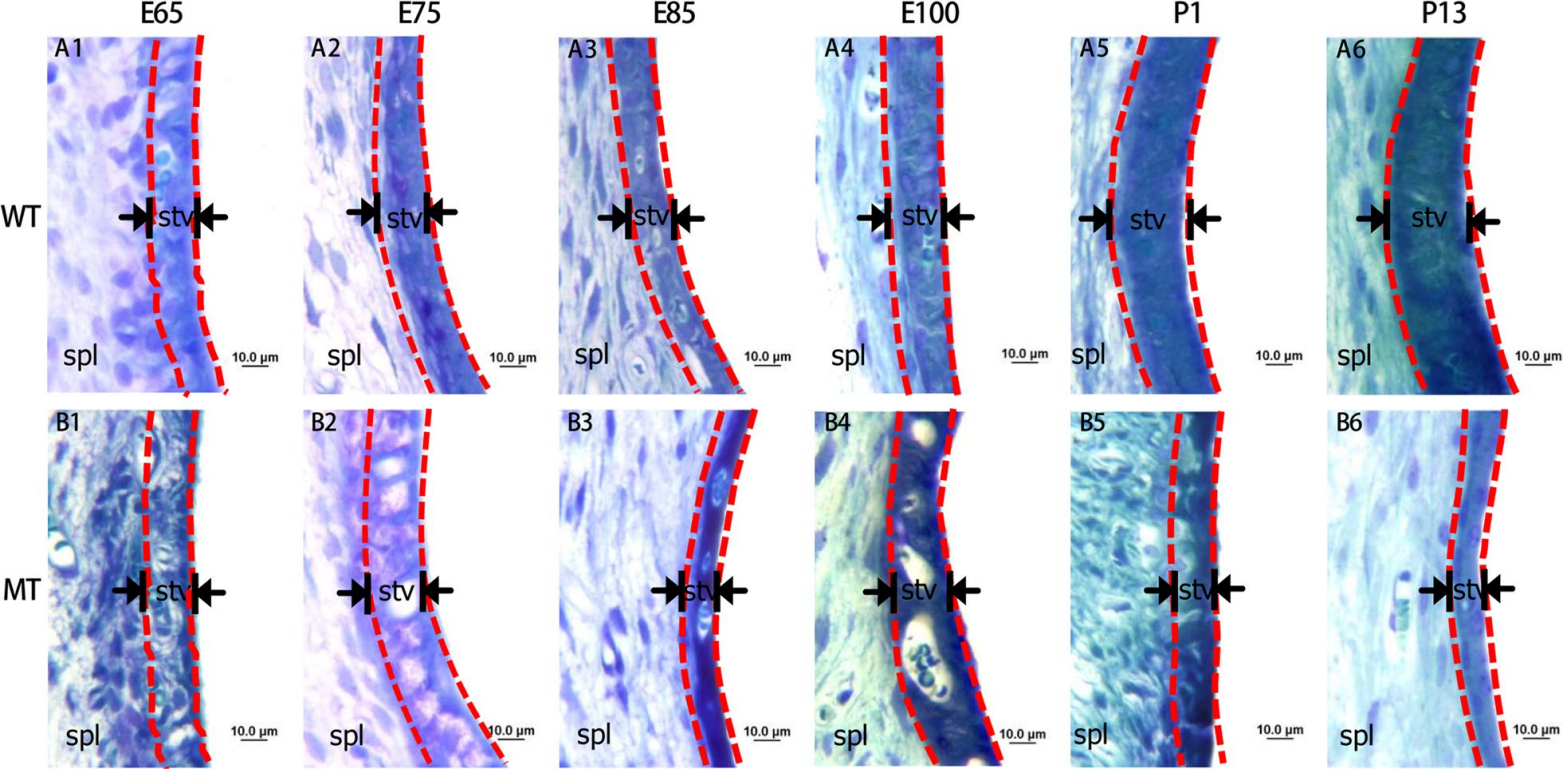
Malformation of the stria vascularis (SV) in developing cochleae of pigs with MITF-M mutation. **A1**–**6** Illustrate the SV in the wild type cochleae from E65 to P13. **B1**–**6** Illustrate the SVs in mutant pigs from E65 to P13. Images show that the SV of MITF-M mutant pigs are remarkably thinner than the normal pigs after E85. All images were taken from the basal turn of cochleae (Wild type, WT; mutant type, MT; spiral ligament, Spl; stria vascularis, stv. Bar = 10 μm)

Semi-thin Sections of the SV: To investigate the development of SV in the MITF-M specific mutation porcine model, we examined the morphology of the SV at different embryonic and postnatal stages (E65, E75, E85, E100, P1 and P13) using light microscopy (LM) and transmission electron microscopy (TEM). The semi-thin sections of SV stained with xylidine blue were observed though LM to monitor the morphogenesis of SV. For the wild-type Rongchang Pig (MITF^+/+^), a dense area could be observed at the lateral wall of the cochlear scala media, but no clear boundary with the adjacent spiral ligament at embryonic Day 65 (E65) was found (Fig. 1A1). The SV became thickened from E65 to E85 and then became increasingly compact from E85 to E100, and matured after E100 (Fig. 1A1–6). For the mutant-type (MITF ^−/−^), the distinctions with the wild-type were showed at E85, when the SV became thinner and looser.

**Fig. 2:**
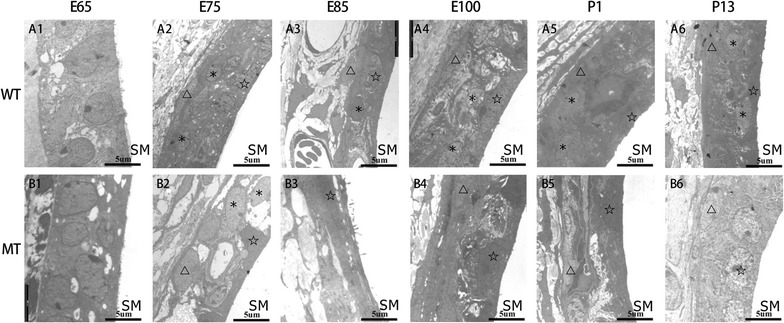
Lack of intermediate cells in the developing stria vascularis (SV) of MITF-M mutant pigs from E65 to P13. Since E75, the SV in the wild type cochleae was typically comprised of three layers: marginal, intermediate and basal cell layer (**A1**–**6**). In contrast, since E85 the SV in mutant type appeared to be extremely disorganized with absence of intermediate cells, and degeneration of marginal and basal cells (**B1**–**6**) (Wild type, WT; mutant type, MT; scala media, SM; marginal cells, pentacles; intermediate cells, asterisks; basal cells, triangles. Bar = 5 μm)

The SV under transmission electron microscopy revealed ultrastructural differences of the developing SV between the MITF^+/+^ and MITF ^−/−^ genotypes (Fig. 2). The intermediate cells (IC) of SV could be identified in both genotypes at E65. For the wild-type, the SV developed three layers at E75, consisting of the marginal cell (MC) layer, the intermediate cell layer and the basal cell (BC) layer. At E85, the marginal cells exhibited numerous microvilli at the apical membrane and extended processes at the basal membrane with centrally located nuclei, while the spindle-shaped basal cells were paralleled to the SV. At E100, marginal cells showed decreased microvilli, flattened nuclei and elongated processes that interacted with the intermediate cells and capillaries. At P1 and P13, the marginal cells were lengthened and connected with each other through tight junctions to form a barrier of the SV after birth. In contrast, the SV of mutant-type appeared totally under developed since E75 when processes at the basal membrane of marginal cells were gradually aggregated and failed to interact with the intermediate cells and mesenchymal capillaries. The intermediate cells began to reduce at E85. After E100, the differences of the SV between two genotypes were more apparent. A discernable boundary was formed between the marginal cell layer and the basal cell layer with only a few intermediate cells embedded in processes at the basal membrane of marginal cells. Subsequently, intermediate cells were rarely observed in the postnatal SV (P1 and P13), leading an extremely thin SV. In summary, the SV of the MITF^−/−^ cochlea was initially under developed and degenerated progressively over time.

### Defective hair cells

We further examined the morphology of the developed organ of Corti at high magnification of LM and the hair cells using scanning electron microscopy (SEM).

**Fig. 3:**
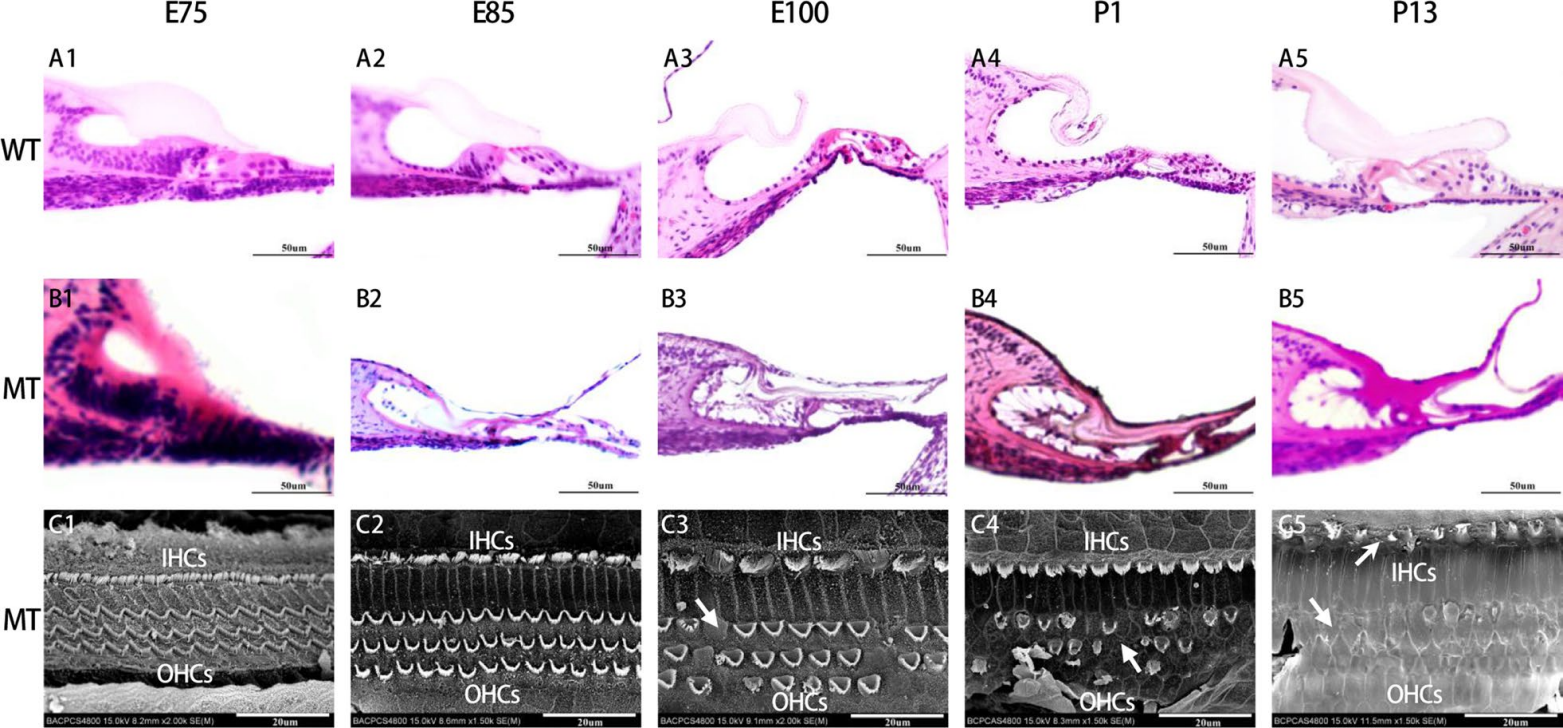
Defected organ of Corti and sensory hair cells in the cochleae of the MITF-M mutant pigs from E75 to P13 (**B1–5**). All images were taken from the basal turn of cochleae. The organ of Corti and sensory hair cells in the cochleae of WT pigs were manifested from E75,E85,E100,P1 and P13 (**A1–5**). The SEM image of hair cells in the cochleae of the MITF-M mutant pigs were manifested in **C1–5**. The degeneration of the organ of Corti in the MT cochlea was manifested from E100 and onwards (**B3**–**5**) as absence of inner and outer hair cells was observed (**C3**–**5**). All images were taken from the basal turn of cochleae. Wild type, WT; mutant type, MT; inner hair cells, IHCs; outer hair cells, OHCs. Bar in **A1**–**B5** = 50 μm, Bar in **C1**–**5** 20 μm

The organ of Corti in MITF^−/−^ embryos was similar to that in MITF^+/+^ embryos between E75 and E85. From E100, the organ of Corti degenerated with loss of sensory epithelia in the MITF ^−/−^ cochlea, when the Reissner’s membrane began to collapse (Fig. 3).

To have a better understanding of the altered structures of hair cells, we observed the hair cells through SEM at E75, E85, E100, P1 and P13 (Fig. 3). It is showed that porcine cochleae were extremely similar with human, as four rows of hair cells arranged along the entire cochlear duct. There were only one row of inner hair cells (IHCs) located in the inner most row toward the central region of the cochlea and three rows of outer hair cells (OHCs) located toward the peripheral region of the cochlea. The hair bundles of OHCs are in a “V”—pattern in the basal and middle turns, whereas in a “C”—pattern in the apical turn. The stereocilia of hair cells lengthened from the basal turn to the apical turn. Until E85, no significant difference was observed between two genotypes. The loss of hair cells and stereocilia bundles in the mutant-type cochleae were manifested and deteriorated since E100.

### Collapsed cochlear duct

**Fig. 4:**
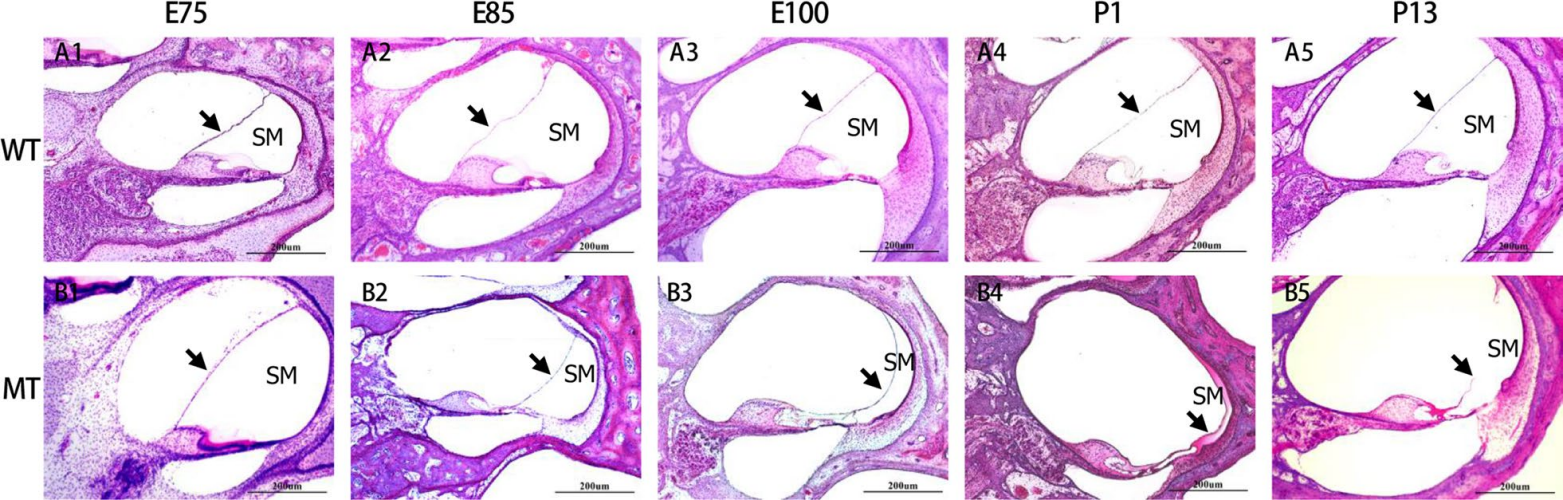
Histopathological changes of cochlear ducts (scala media) in the MITF-M mutation pigs. **A1**–**5** Illustrate the normal development of the scala media (SM) in wild-type pigs from E75 to P13. **B1**–**5** illustrate the progressive collapsion of the cochlear ducts from E75 to P13. Reissner’s membrane completely descended onto the organ of Corti in the E100 MT cochlea (**B3**). All images were taken from the basal turn of cochleae (Wild type, WT; mutant type, MT; scala media, SM. Bar = 200 μm)

To investigate the time course of cochlear duct formation along with the under developed SV, we compared the mid-modiolar cross celloidin sections through the cochlea in both genotypes at E75, E85, E100, P1 and P13 (Fig. 4). At E75 and E85, the morphology and size of the cochlear duct appeared similar in two genotypes that the volume of the cochlear duct gradually increased with elongation of the SV. The inner spiral sulcus space was first spotted at E75. At E85, the tunnel of Corti and the space of Nuel were distinguishable, while the amorphous tectorial membrane developed to lay over the greater epithelial ridge. By E100, the cochlear gross morphology of wild-type Rongchang pig had matured, whereas the volume of cochlear duct had shrunk and the fine structure of the organ of Corti had deteriorated due to the collapse of Reissner’s membrane in the mutant-type. These pathological changes occurred similarly at all cochlear turns of the mutant-type.

**Fig. 5:**
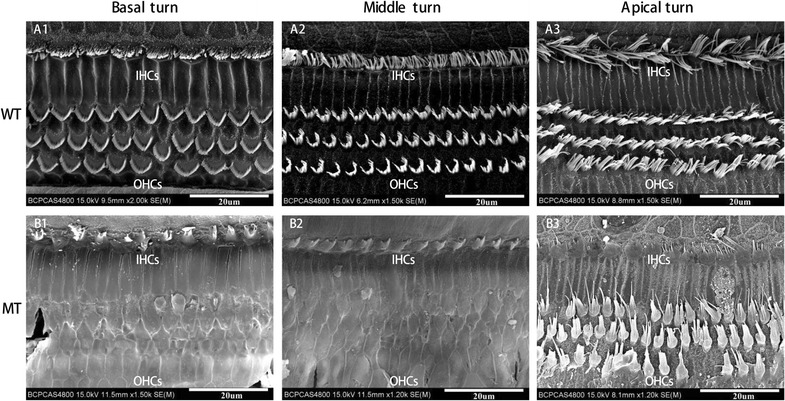
Missing stereocilias (arrow) of inner and outer hair cells in the cochleae of the postnatal MITF-M mutation pigs shown by a scanning electron microscope. The stereocilias of hair cells in inner hair cells (IHCs) and outer hair cells (OHCs) of WT (**A1–3**) and MT (**B1–3**) were manifested in Basal turn, Middle turn, and Apical turn, respectively. The stereocilias of hair cells in basal turn of MT cochlea was most severe. Wild type, WT; mutant type, MT; inner hair cells, IHCs; outer hair cells, OHCs. Bar = 20 μm

Figure 5 showed the different degree of degeneration of inner and outer hair cells in the cochleae of the postnatal MITF^−/−^ mutation pigs. The loss of stereocilias of hair cells, both inner hair cells (IHCs) and outer hair cells (OHCs), were most severe in the basal turn.

### SGC degeneration

**Fig. 6:**
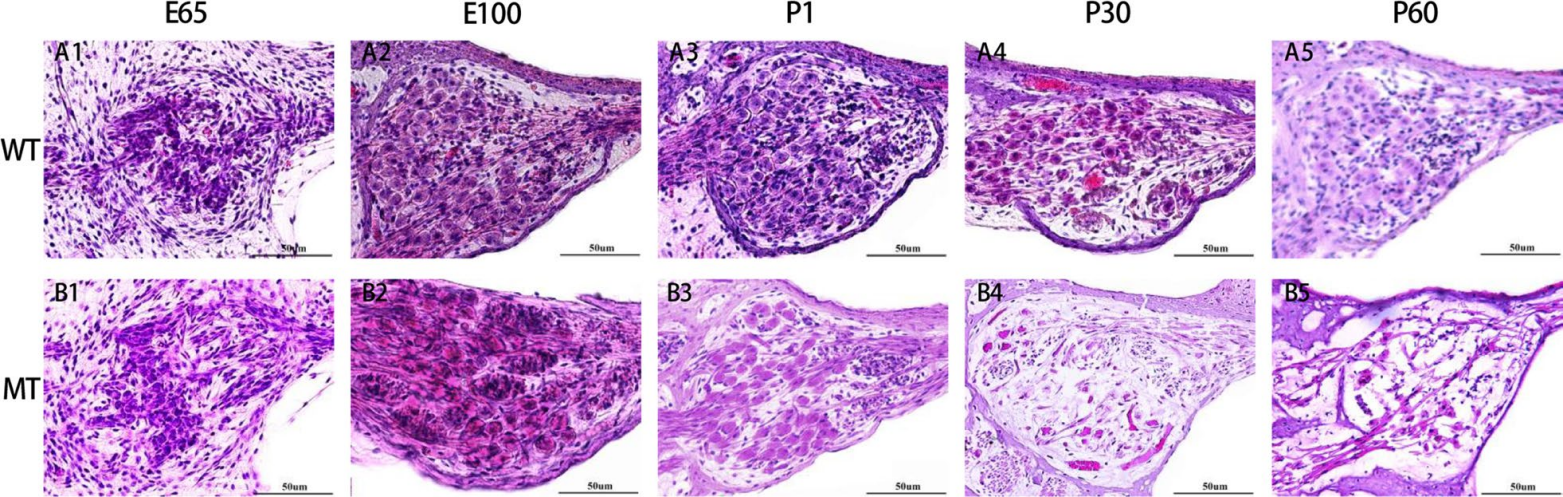
Delayed degeneration of spiral ganglion cells in the cochleae of the WT (**A1–5**) and MITF-M mutation pigs (**B1–5**) at E65, E100, P1, P30 and P60, respectively. The spiral ganglion cells began to decrease apparently at P30 (**B4**) with other changes including swollen cells, shrunken nuclei and increased intercellular gaps. All images were taken from the basal turn of cochleae. Wild type, WT; mutant type, MT. Bar = 50 μm

In addition, we observed cochlear SGCs at high magnification of LM to study their pathologic changes at E65, E100, P1, P30 and P60. The results (Fig. 6) showed no distinct difference of cochlear spiral ganglions between the mutant-type and the wild-type Rongchang pig from E65 to P1. However, the number of SGNs decreased significantly at P30 with other changes including swollen cells, shrunken nuclei and increased intercellular gaps.

**Fig. 7:**
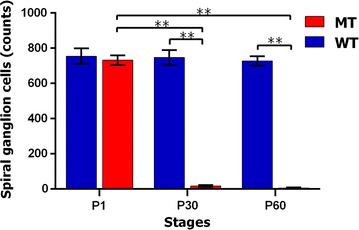
MITF mutation caused a significant decrease of the number of spiral ganglion cells (SGCs) after P30. The SGCs were counted in the MITF^+/+^ and MITF^−/−^ cochleae at P1, P30 and P60. Most SGCs of MITF^−/−^ cochleae were lost since P30. The data were presented as the mean ± SD (n = 6). All SGCs were counted from the basal turn of cochleae. Wild type, WT; mutant type, MT. (**P < 0.01)

The number of SGNs in the MITF^−/−^ pigs was counted at different stages (P1, P30 and P60) after hair cell loss. The SGNs in the basal turns of each slice were counted at high magnification (400 ×). The results were presented as mean ± SD. As shown in Fig. 7, the number of SGNs of MITF^−/−^ cochleae 732.0 ± 27.7 at P1, 17.0 ± 4.7 at P30 and 6.2 ± 3.0 at P60. There was a significant difference between P1 with P30 and P60 (One-way ANOVA, P < 0.01 in P1 vs. P30 and P1 vs. P60) and no significant difference after P30 (One-way ANOVA, P > 0.01). There was no significant difference of the SGNs at P1 between the mutant and wild types (Student’s t test). However, the SGNs number in MITF^−/−^ cochleae at P30 and P60 were both significantly less than that in MITF^+/+^ cochleae (both P < 0.01).

## Discussion

It is known that hearing loss in the WS is due to the abnormal proliferation, survival, migration, or differentiation of neural crest-derived melanocytes in inner ear which are situated in the middle layer of the SV called intermediate cells. Histopathologic findings of WS both in patients and animal models indicate that the primary alteration is loss of the intermediate cells in the SV, whereas all the other alterations are secondary, including atrophy of the organ of Corti, collapse of the endolymphatic space and degeneration of the SGCs [18–20]. In our previous studies, we had described the morphological changes found in the postnatal cochlear of Rongchang pigs induced by MITF-M mutation [17]. In this study, we further examined the morphological changes in embryonic stages in this porcine model. Our results revealed that the hearing loss in the MITF-M mutant pigs are primarily caused by the malformation of the SVs and the progressive reduction of the cochlear duct and the degeneration of sensory epithelium of the developing inner ears. Our findings on this MITF-M mutant pig model is consistent with previous reports.

In the MITF^−/−^ porcine cochleae, the reduction and absence of intermediate cells in the SV was observed since E85, which indicates that melanocytes in MITF-M mutation pigs are still able to migrate to the cochlea, but have a deficiency in survival during embryogenesis. Subsequently, the Reissner’s membrane descended and cochlear duct finally collapsed with apoptosis of hair cells by E100. Unlike other altricial species that cochleosaccular degenerations were mainly observed at postnatal period, the pig models and humans showed a similar pattern that cochlear disorder happened embryonic stages [21–23]. Cochlear implant, which mainly depend on the survival of cochlear SGCs, is an effective treatment for deaf patients with WS. It is confirmed that outcomes of cochlear implant in WS children are similar to other non-syndromic SNHL children [24–26]. It is also suggested that patients who received early intervention show a better hearing and language skills [27]. However few studies have identified the intervention time and the outcome of the cochlear implants. It is manifested in the current study that the SGNs in the MITF-M mutation pigs began to degenerate gradually one month after birth. However, the morphology of the survival SGNs seemed to be normal. The number of SGCs in MITF^−/−^ cochleae significantly decreased since P30, which had no significant difference among MITF^+/+^ cochleae at P1, P30 and P60. Delayed degeneration of the SGNs in the MITF^−/−^ pigs indicates that cochlear implant is an effective option for auditory rehabilitation of patients with WS and it appears that the earlier of cochlear implant, the better outcomes. This porcine model may also be used to study whether the SGNs in WS patients would remain after the cochlear implant.

## Conclusions

In summary, the malformation of the SV was the primary defect in the cochleae of the MITF-M mutation porcine model. The neural crest-derived melanocytes might migrate to the developing SV but fail to survive leading to malformation of the marginal and basal cells. Subsequently, the cochlear duct collapsed and hair cells degenerated are the secondary cochleosaccular degeneration caused by the MIFT mutation. However, no significant change was found in the SGNs of MITF^−/−^ pigs until one month after birth. This result suggests that WS patients should be good candidates for cochlear implant. Earlier receiving cochlear implant, better outcomes after the surgery. This MITF-M mutation porcine model will provide a crucial animal model for further research on cochlear implant, cell therapy in patients with congenital hereditary deafness.

## Abbreviations

WS: Waardenburg syndrome
E85: embryonic 85 days
NC: neural crest
P30: postnatal 30 days
MITF: microphthalmia-associated transcription factor
bHLH-LZ: basic helix–loop–helix leucine zipper
MITF-M: melanocyte-specific isoforms
SV: stria vascularis
SGNs: sprial ganglion cells
PLA: People’s Liberation Army
EDTA: ethylene diamine tetraacetic acid
HE: hematoxylin–eosin
TEM: transmission electron microscopy
SEM: scanning electron microscopy
SD: standard deviation
MT: mutate type
WT: wild type
ANOVA: analysis of variance
LM: light microscopy
IC: intermediate cells
MC: marginal cell
BC: basal cell
IHCs: inner hair cells
OHCs: outer hair cells
